# The relationship between workplace violence and turnover intention among psychiatric nurses: the mediating roles of ward atmosphere and social distance

**DOI:** 10.3389/fpubh.2026.1828081

**Published:** 2026-06-12

**Authors:** Deyu Liu, Junyu Liu, Yuan Luo, Liping Zhao, Haiye Ran, Linjing Zhang, Yusheng Tian, Jianjian Wang

**Affiliations:** 1 Xiangya School of Nursing, Central South University, Changsha, China; 2 The Second Xiangya Hospital of Central South University Mental Health Institute, Changsha, China; 3 School of Nursing, Capital Medical University, Beijing, China; 4 The Second Xiangya Hospital of Central South University, Changsha, China

**Keywords:** psychiatric nurses, social distance, turnover intention, ward atmosphere, workplace violence

## Abstract

**Background:**

Psychiatric nurses face high turnover rates and increasing levels of turnover intention. Workplace violence is associated with turnover intention among psychiatric nurses. Social exchange theory suggests that ward atmosphere and social distance may play a significant role in this process. However, the mechanisms underlying this relationship remain unclear.

**Objective:**

To explore how workplace violence influences nurses' turnover intention through ward atmosphere and social distance, and to provide strategies for promoting nurses' wellbeing.

**Methods:**

A questionnaire survey was conducted among 1,485 psychiatric nurses recruited through the Mental and Psychological Care Alliance from September to October 2024. Variables were measured using a demographic questionnaire, a turnover intention item, the Workplace Violence Scale, the Ward Atmosphere Scale, and the Social Distance Scale. Statistical analysis and model construction and validation were performed using SPSS 29.0 software.

**Results:**

Ward atmosphere was negatively correlated with turnover intention (*r* = −0.423), social distance (*r* = −0.340), and workplace violence (*r* = −0.307). Turnover intention was positively correlated with both social distance (*r* = 0.221) and workplace violence (*r* = 0.214). Workplace violence was positively correlated with social distance (*r* = 0.159). Workplace violence directly affected turnover intention among psychiatric nurses (β = 0.181, *p* < 0.001). The workplace violence also indirectly affects turnover intention among psychiatric nurses through the simple mediating effect of ward atmosphere (β = 0.232, *p* < 0.001) and social distance (β = 0.010, *p* < 0.001). And the sequential mediating effect of ward atmosphere and social distance between workplace violence and turnover intention is also significant *(*β = 0.017, *p* < 0.01). Additionally, that ward atmosphere and social distance totally mediated the link between workplace violence and turnover intention (indirect effect = 0.259, 95% CI: 0.207–0.313), explaining 58.863% of the total effect.

**Conclusion:**

This study suggests that we should pay attention to the influencing factors of the turnover intention and take effective measures. It is imperative to mitigate the occurrence of workplace violence within the ward. Additionally, we should cultivate a positive ward atmosphere, enhance the attitudes of psychiatric nurses toward their patients, and diminish expectations of social distance. These actions may reduce nurse turnover.

## 1 Introduction

The global psychiatric nursing shortage has emerged as a critical challenge (1). This shortage exacerbates nurses' workload and diminishes job satisfaction, thereby compromising nursing quality and patient outcomes, a challenge further compounded by elevated nurse turnover rates (2–4). China has an average of 3.77 psychiatric nurses per 100,000 residents (5), a rate significantly lower than that of upper-middle-income countries (5.1 per 100,000) and high-income countries (29 per 100,000) (1). The occurrence of turnover behavior is a gradual process of intent-to-leave accumulation (6). High turnover rates not only increase recruitment and training costs for hospitals but also lead to a waste of nursing educational resources. Moreover, turnover intentions (TI) may increase the stress and burnout of remaining nurses, perpetuating a vicious cycle of TI (7, 8). Since TI is a reliable predictor of actual employee turnover, as demonstrated by Namin et al. (9), it is crucial to delve into the factors that shape the TI of psychiatric nurses. This exploration is essential for addressing staffing shortages and reducing organizational costs.

Previous research has found that workplace violence (WPV) is a strong predictor of nurses' TI (10). Furthermore, psychiatric wards represent high-risk environments for WPV, with an umbrella review reporting that patient violence accounts for 78% of the WPV events experienced by psychiatric nurses (11)—a prevalence rate significantly higher than that in other wards. Therefore, it is particularly important to explore the potential mechanism between WPV and TI among psychiatric nurses. Although some studies have proved that WPV can positively predict TI, and preliminarily revealed the correlation between the two, research on the mediating mechanisms through which WPV affects TI is limited. Meanwhile, empirical research on how WPV affects TI from a multi-foci perspective remains notably lacking. This paper introduces ward atmosphere and social distance to analyze the relationship between WPV and TI, aiming to reveal the complex mechanisms involved among psychiatric nurses.

**Figure 1:**
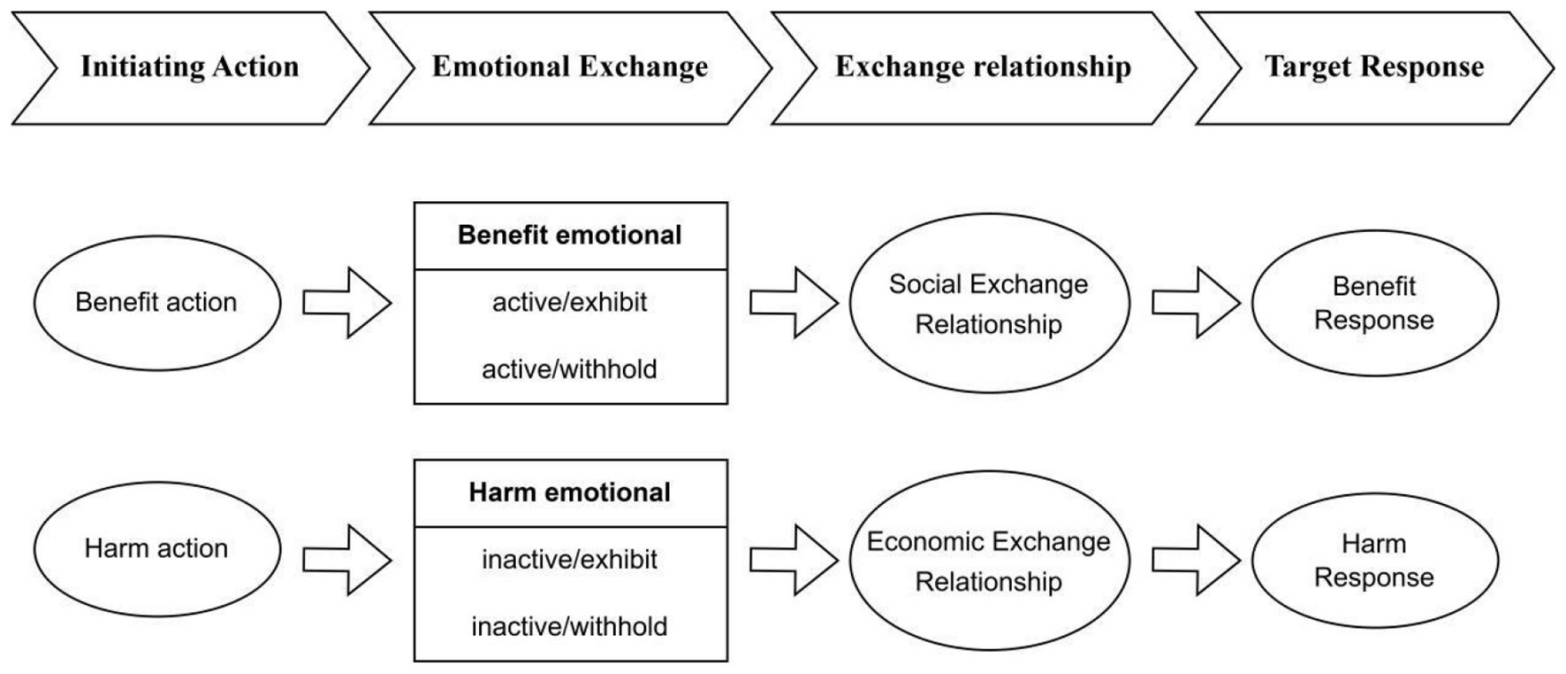
SET model of the two dimensions of emotional social exchange.

Social exchange theory (SET) continues to be one of the most influential theoretical frameworks for explaining workplace behavior (12) (see Figure 1). At the core of SET is the principle of reciprocity, which can manifest in both positive (i.e., respond to positive treatment with positive treatment) or negative (i.e., respond to negative treatment with negative treatment) (13). Building on this concept, homologous reciprocity (14) emphasizes retaliatory norms in negative exchanges, prioritizing the return of harm rather than benefit. Furthermore, Cropanzano et al. (15) constructed a two-dimensional taxonomy of homologous reciprocity, which included four quadrants—from “active/exhibit” on the top to “inactive/withhold” on the bottom. A critical extension of SET concerns the nature of transactions. Martin and Harder (16) added emotional theory to SET, distinguishing between tangible economic exchanges and symbolic socio-emotional exchanges. Ahmad et al. (17) refined this framework by conceptualizing “psychological exchange” as a form of “inactive/withhold” reciprocity. To capture the complexity of multi-stakeholder influences, Lavelle et al. (18) introduced a multi-foci perspective through their target similarity model, which demonstrated how heterogeneous exchange relationships collectively shape employees' cognitive evaluations, interpersonal dynamics, and behavioral responses.

### 1.1 WPV and TI

WPV refers to any act, incident, or behavior in which nurses are attacked, threatened, or humiliated by patients, relatives, or visitors in professional settings, including physical, verbal, and psychological violence (11). Emerging evidence suggested that verbal and psychological violence may have stronger associations with turnover intention than physical violence (19). Statistics on Hospital Workplace Violence in China 2000–2020 demonstrated that patient-derived violence constituted the majority (95.4%) of all incidents (20). Existing studies have reported that WPV affects the wellbeing of psychiatrists and nurses, hinders psychiatric workforce stability, and is associated with increased TI in sampled populations (21–23). In line with SET, employees tend to reciprocate positive workplace experiences with enhanced engagement and productive behaviors. Conversely, they may exhibit a downward adjustment in their attitudes and behaviors when subjected to unfavorable treatment. WPV, as an unfavorable treatment in the workplace of psychiatric nurses, can lead to negative interactions, which in turn lead to unfavorable work behaviors and increased TI among nurses. Thus, we hypothesize the following:

Hypothesis 1. WPV is positively associated with high TI among psychiatric nurses.

### 1.2 Mediating effect of ward atmosphere

The ward atmosphere, developed specifically for psychiatric settings (24), is utilized to characterize the psychosocial climate of inpatient units (25). The Essen Climate Evaluation Schema (EssenCES) measures three dimensions of ward atmosphere, including patient cohesion and mutual support, experienced safety and therapeutic hold (26). The ward atmosphere represents a dynamic interplay among architectural, organizational, staff, and patient characteristics (27). This complex interplay has the potential to shape the emotions, behaviors, and self-perception of both patients and staff (28). WPV has a detrimental impact on psychiatric nurses' perceptions of the ward atmosphere. The experimental study proved that the introduction of Safewards reduced the occurrence of WPV within 1 year, and the median scores of the three dimensions of ward atmosphere perceived by nurses increased from 8, 7.5 and 15 to 12, 13 and 18, respectively (29). Owing to the negative reciprocity norm of SET (14), WPV triggers negative reciprocity, prompting nurses to reduce their engagement with organizational resources and perceive the ward atmosphere more negatively (29). Prolonged exposure to a negative ward atmosphere may exacerbate nurses' emotional exhaustion, which could potentially lead to voluntary turnover as a mechanism to halt depletion of personal resources (30). Therefore, we hypothesize the following:

Hypothesis 2. Ward atmosphere plays a mediating role between WPV and TI among psychiatric nurses.

### 1.3 Mediating effect of social distance

Social distance is a psychological construct reflecting preferred interpersonal closeness in social interactions (31). It is commonly measured using the Social Distance Scale (SDS), which assesses willingness to interact with individuals with mental illness in various social roles (32). Among mental health professionals, higher social distance has been associated with more negative attitudes and lower therapeutic alliance (31, 74). It serves as a measurable indicator of individuals' attitudes toward psychiatric patients (33). Adverse social experiences, such as increased exposure to WPV, significantly increase psychiatric nurses' social distance from patients (34). The emotional exchange within this process—characterized as inactive/withheld and implicit—differs from the ward atmosphere-related exchange process mentioned earlier (15). On the basis of the SET of psychological transactions, the experience of WPV increases nurses' implicit bias toward psychiatric patients. To safeguard their own resources, nurses may protect their own resources through emotional exclusion, which in turn widens their expected social distance from psychiatric patients. In addition, social distance has a negative impact on medical students' choice of employment in psychiatry (35). A previous study reported that positive attitudes toward psychiatric patients among mental health staff are negatively correlated with TI (36). In contrast, WPV-triggered emotional exclusion may escalate through negative reciprocity, culminating in greater TI. Therefore, we hypothesize the following:

Hypothesis 3. Social distance plays a mediating role between WPV and TI among psychiatric nurses.

### 1.4 Serial mediating effect of ward atmosphere and social distance

Workplace relationships are complex and multifaceted. Methot et al. (37) introduced the term “multiplex” workplace relationships, and the exchanges within these complex relationships form a “transaction chain” (17, 38). However, the mechanisms through which the ward atmosphere (rooted in environmental resource exchange) and social distance (arising from psychological exchange) influence the interplay between WPV and nurses' TI remain underexplored. Studies have shown that the working environment of psychiatric nurses can affect their attitudes toward patients (39, 40), and that negative environmental perceptions can increase social distance from others (41). Based on Social Exchange Theory (SET) and the multi-foci perspective, employees maintain distinct social exchange relationships with different foci, such as the broader organization (or its climate) and specific individuals (e.g., patients). Workplace violence first damages the exchange with the organizational environment, reflected in a negative ward atmosphere. This depleted organization-level exchange then “spills over” to impair the exchange with the specific target of patients, thereby increasing social distance. Therefore, we hypothesize the following:

Hypothesis 4. Ward atmosphere and social distance could serially mediate the relationship between WPV and TI among psychiatric nurses.

### 1.5 Research objectives and hypotheses

**Figure 2:**
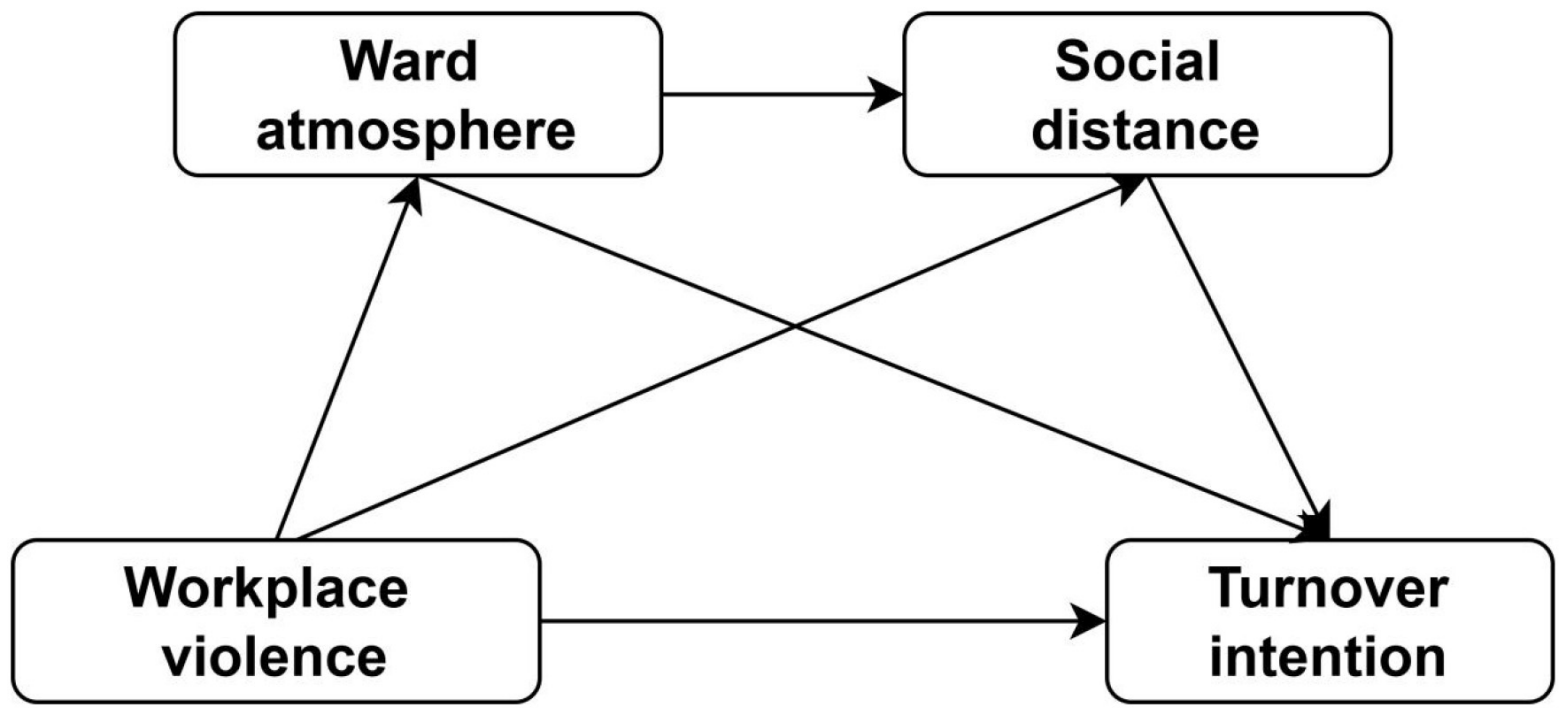
Hypothesized serial mediation model of workplace violence, ward atmosphere, social distance, and turnover intention.

Guided by SET, this study delved into the impact of workplace violence on the turnover intentions of psychiatric nurses, with a particular focus on the mediating roles of ward atmosphere and social distance. On the basis of the findings of previous studies, we constructed the research framework diagram in Figure 2 and proposed the following hypotheses:

Hypothesis 1. WPV is positively associated with high TI among psychiatric nurses

Hypothesis 2. Ward atmosphere plays a mediating role between WPV and TI among psychiatric nurses.

Hypothesis 3. Social distance plays a mediating role between WPV and TI among psychiatric nurses.

Hypothesis 4. Ward atmosphere and social distance played a serially mediating role in the relationship between WPV and TI among psychiatric nurses.

## 2 Method

### 2.1 Design, setting, and participation

This study employed a cross-sectional research design. From September to October 2024, questionnaires were distributed via WeChat to members of the Mental and Psychological Care Alliance of the National Center for Disorders. Participants filled out the questionnaires anonymously by scanning the QR code. The completed questionnaires and signed informed consent forms were returned to our researchers. The sample size was calculated via the formula $N=\frac{\mu_{\alpha }^{2}•\pi •\left(1-\pi \right)}{\delta^{2}}$ (π = prevalence of nurses' turnover intentions, π = 20% (42), μ_α_ = 2.58, δ = 0.03), and the minimum sample size was 1,184. Taking into account a potential dropout rate of 10%, a sample size of at least 1,316 participants would be required. Finally, 1,485 questionnaires were distributed, and 1,460 were returned (response rate: 98.32%). After excluding 123 invalid questionnaires because of missing data, completion times of less than 1 min, or logical inconsistencies (such as having 1 year of working experience matching a senior professional title), 1,337 valid questionnaires were retained. The average age of participants was 34.9 ± 7.98 years, and most were female (*n* = 1,134, 84.8%).

The inclusion criteria were as follows: (a) voluntarily participated in this study and provided informed consent; and (b) were on-duty psychiatric nurses. The exclusion criteria were as follows: (a) new nurses (≤3 months), (b) standardized training nurses (a newly graduated nurse participating in mandatory structured training) and refresher training nurses (an experienced nurse pursuing specialized skill enhancement through short-term elective training).

### 2.2 Measures

#### 2.2.1 Demographic characteristics

The following demographic data were collected: age, gender, marital status, educational level, and monthly income. Additionally, occupational information, including professional title, hospital level, working years, employment type, training history (such as emotional regulation, violence prevention, and protective constraints), and ward type, was also collected.

#### 2.2.2 Turnover intention

In the present study, following previous studies (43, 44), a single 5-point Likert-scale item was used to evaluate nurses' TI. This item presented respondents with five response options, ranging from “1” (indicating “very unwilling”) to “5” (representing “very willing”).

#### 2.2.3 Workplace violence

The scale was developed by the Chinese scholar Wang (45) to assess the frequency of WPV in the past year. It comprises 5 items utilizing a 4-point Likert-type response format, with options ordered hierarchically by incident frequency. The score follows an ordinal system: 0 points for no violence, 1 point for 1 incident, 2 points for 2–3 incidents, and 3 points for≥4 incidents. The total scale score is calculated by summing the item-level scores (range: 0–15). A higher total score indicates a greater frequency of WPV. The scale also categorizes the frequency of WPV as follows: zero frequency (0 points), low frequency (1–5 points), medium frequency (6–10 points), and high frequency (11–15 points). In the present study, the Cronbach's α for the WPV Scale was 0.857.

#### 2.2.4 Ward atmosphere

The Chinese version of the Essen Climate Evaluation Schema (EssenCES) by Schalast et al. (26) was used in this study. The scale was translated and validated in Chinese psychiatric nurses and patients by Liu et al. (46). The EssenCES is a 15-item questionnaire designed to assess the social and therapeutic atmosphere in psychiatric wards in three dimensions: patient cohesion and mutual support, experienced safety, and therapeutic hold. The respondents rated each item on a 5-point Likert scale, with options ranging from 0 (not at all) to 4 (very much). Total scores are derived by summing item responses, yielding a composite range of 0–60. High scores on the EssenCES suggest a positive ward atmosphere. In the present study, the internal consistency coefficient of the scale was found to be 0.848.

#### 2.2.5 Social distance

The Social Distance Scale (SDS) was developed by Link et al. (32) and translated into Chinese by Li (47). It was used to assess nurses' social distance from psychiatric patients. The questionnaire comprises 6 items, each scored on a 5-point scale from “1” (very willing) to “5” (very unwilling). A higher score indicates greater reluctance to engage with psychiatric patients. The internal consistency coefficient of this scale was 0.950.

### 2.3 Ethics considerations

Ethical approval for the study was granted by the Ethics Committee of the Second Xiangya Hospital of Central South University (2024–060). This research was performed in accordance with the Declaration of Helsinki. All participants provided written informed consent prior to enrollment, and the study protocol was approved by the Institutional Review Board (IRB). Personal data were anonymized to ensure confidentiality.

### 2.4 Data analysis

SPSS 29.0 was used in this study for data collation and analysis. The data followed an approximately normal distribution. The measurement data are expressed as the means ± standard deviations *(M* ± *SD)*, and the count data are expressed as frequencies and percentages. Univariate analysis was conducted via the chi-square test and the Kruskal–Wallis test. Pearson correlation analyses were performed to examine the relationships between key variables: WPV, TI, ward atmosphere, and social distance.

Prior to statistical analysis, common method bias was assessed using Harman's single-factor test. The results showed that the unrotated first factor explained only 22.75% of the total variance, below the 40% threshold (48). Confirmatory factor analysis (CFA) was conducted to evaluate factor loadings, composite reliability (CR), and average variance extracted (AVE) for convergent validity assessment. The variance inflation factor (VIF) was calculated to detect multicollinearity among continuous independent variables. Multiple linear regression was conducted to examine the unique contributions of WPV, ward atmosphere, and social distance to TI among psychiatric nurses. TI was set as the dependent variable, and WPV, ward atmosphere, and social distance were entered simultaneously as independent variables using the forced entry (Enter) method. Mediation analyses were carried out via the SPSS PROCESS v.4.0 macro (Andrew F. Hayes, The Ohio State University, Columbus, USA) (49) to estimate the total, indirect, and chain indirect effects. A total of 5,000 bootstrapping resamples were used to generate 95% confidence intervals (CIs) for two-tailed tests. In Model 6, we specified WPV as the independent variable, TI as the dependent variable, and ward atmosphere followed by social distance as chain mediators in the pathway. The statistical significance level α was set to 0.05.

## 3 Results

### 3.1 Sample characteristics

Participants were recruited from Hunan, Anhui, Guangdong, Jilin, Gansu, Hubei, Fujian, Shanxi, Hebei, Yunnan, Jiangxi, Zhejiang, Henan, Liaoning, Sichuan, Beijing, Xinjiang, Guangxi, and Inner Mongolia, China. The average length of nursing work experience was 12.88 ± 8.84 years. Among the 1,337 participants, most had an undergraduate education (*n* = 955, 71.4%), and were married (*n* = 1,023, 76.5%). A total of 47.6% (*n* = 637) reported a monthly income of 5,001–10,000 yuan, 85.1% (*n* = 1,138) held a junior professional title, and 68.9% (*n* = 921) worked in tertiary hospitals. Off-staff nurses constituted 62.7% (*n* = 840) of the sample. High training coverage was observed for emotional management (79.1%), violence prevention (94.9%), and protective constraints (98.7%). Most nurses work in close wards (77.6%). Detailed information is presented in Table 1.

**Table 1:** Sample demographic characteristics (*N* = 1,337).

Variables	*N*	%	TI (M ±SD)	P
Gender				0.379
Male	203	15.2	2.40 ± 0.90	
Female	1,134	84.8	2.34 ± 0.88	
Age (years old)				< 0.001
21–30	447	33.5	2.45 ± 0.90	
31–40	581	43.4	2.40 ± 0.84	
41–50	242	18.1	2.17 ± 0.87	
51–60	67	5.0	2.00 ± 0.90	
Educational level				0.756
Below the undergraduate	362	27.1	2.17 ± 0.86	
Undergraduate	955	71.4	2.36 ± 0.88	
Master and above	20	1.5	2.30 ± 0.80	
Marital status				0.175
Single	275	20.6	2.43 ± 0.89	
Married	1,023	76.5	2.33 ± 0.87	
Divorced	38	2.8	2.42 ± 1.08	
Widowed	1	0.1	1.00	
Monthly income (Yuan)				< 0.001
< 3,000	122	9.2	2.61 ± 0.87	
3,000–5,000	440	32.9	2.48 ± 0.86	
5,001–10,000	637	47.6	2.28 ± 0.87	
10,001–20,000	133	9.9	2.08 ± 0.85	
>20,000	5	0.4	2.00 ± 0.70	
Professional title				<0.001
Primary title	1,138	85.1	2.42 ± 0.87	
Intermediate title	170	12.8	1.98 ± 0.82	
Senior title	29	2.1	1.90 ± 0.67	
Working hospital level				0.118
Primary-level	10	0.7	2.20 ± 0.91	
Secondary-level	406	30.4	2.41 ± 0.88	
Tertiary-level	921	68.9	2.36 ± 0.88	
Working years				<0.001
< 10	538	40.2	2.41 ± 0.87	
10–19	509	38.2	2.43 ± 0.87	
20–29	193	14.4	2.19 ± 0.85	
≥30	97	7.2	1.98 ± 0.87	
Employment type				<0.001
In-staff	497	37.2	2.19 ± 0.86	
Off-staff	840	62.7	2.45 ± 0.87	
Training history				
Emotional regulation				**<0.001**
Yes	1,058	79.1	2.27 ± 0.85	
No	279	20.9	2.69 ± 0.88	
Violence prevention				0.003
Yes	1,269	94.9	2.34 ± 0.87	
No	68	5.1	2.66 ± 0.97	
Protective constraints				0.479
Yes	1,319	98.7	2.35 ± 0.88	
No	18	1.3	2.50 ± 0.70	
Ward type				0.052
Close	1,037	77.6	2.38 ± 0.88	
Open	300	22.4	2.27 ± 0.81	

M, mean; SD, standard deviation. Professional title: Primary title, including nurses and nurse practitioners.

Intermediate title included nurses-in-charge. The senior title included co-chief nurses and chief nurses. Level of working hospital: Primary-level hospitals are community-based healthcare institutions that directly serve local residents. Secondary-level hospitals are regional medical centers that provide services across multiple communities.

Tertiary-level hospitals are large-scale institutions offering specialized care across provinces, municipalities, and even nationwide. Employment form: In-staff refers to nurses who hold a permanent establishment position within the public healthcare system. Off-staff refers to nurses employed on a contractual or temporary basis without bianzhi status, which typically denotes permanent employment with pension benefits and job security in Chinese public institutions. Ward type: A closed ward is a psychiatric inpatient unit with restricted access (e.g., locked doors) for patients. The open ward allows voluntary admission for stable patients who retain autonomy (e.g., movement within the facility).

Gender (1 = male, 2 = female); Age (0 = 21–30, 1 = 31–40, 2 = 41–50, 3 = 51–60); Educational level (1 = Below the undergraduate, 2 = Undergraduate, 3 = Master and above); Marital status (1 = Single, 2 = Married, 3 = Divorced, 4 = Widowed); Monthly income (0 = < 3,000, 1 = 3,000–5,000, 2 = 5,001–10,000, 3 = 10,001–20,000, 4 = >20,000); Professional titles (0 = Primary title, 1 = Intermediate title, 2 = Senior title); Working hospital level (0 = Primary-level, 1 = Secondary-level, 2 = Tertiary-level); working years (0 = < 10, 1 = 10–19, 2 = 20–29, 3 = ≥30); Employment type (1 = In-staff, 2 = Off-staff); Emotional regulation Training (1 = Yes, 2 = No); Violence prevention Training (1 = Yes, 2 = No); Protective constraints Training (1 = Yes, 2 = No); Ward-specific type (1 = Close, 2 = Open). Bold p-values indicate statistical significance (*p* < 0.05).

### 3.2 Assessment of reliability and validity

As shown in Table 2, all multi-item scales demonstrated acceptable reliability (Cronbach's α > 0.80). CFA showed that all factor loadings exceeded 0.50. CR values all exceeded 0.70, and AVE values all exceeded 0.50, supporting convergent validity (50). TI was measured by a single item, so CR and AVE were not calculated. VIF values for all independent variables were below 2, indicating no significant multicollinearity.

**Table 2:** Reliability, convergent validity, and multicollinearity (*N* = 1,337).

Variable	Cronbach's α	VIF	CR	AVE	Factor loadings range
WPV	0.857	1.12	0.840	0.518	0.549~0.857
Social distance	0.950	1.08	0.848	0.502	0.496~0.841
Ward atmosphere	0.832	1.25	0.952	0.868	0.862~0.989

CR, composite reliability; AVE, average variance extracted; VIF, variance inflation factor.

### 3.3 Preliminary analyses

Table 3 shows correlations, means, and standard deviations for main variables. Ward atmosphere was significantly negatively correlated with TI (*r* = −0.423, *P* < 0.001), social distance (*r* = −0.340, *P* < 0.001), and WPV (*r* = −0.307, *P* < 0.001). TI was positively associated with both social distance (*r* = 0.221, *P* = 0.003) and WPV (*r* = 0.214, *P* = 0.004). WPV also showed a weak positive correlation with social distance (*r* = 0.159, *P* = 0.047).

**Table 3:** Correlation matrix for the variables of the structural equation model (*N* = 1,337).

Variable	Mean ±SD	1	2	3	4
1. TI	2.35 ± 0.88	1			
2. WPV	5.41 ± 4.29	0.214^**^			
3. Ward atmosphere	37.51 ± 6.81	−0.423^**^	−0.307^**^		
4. Social distance	20.5 ± 3.78	0.221^**^	0.159^**^	−0.340^**^	1

WPV, workplace violence; TI, turnover intention.

^**^P < 0.01.

### 3.4 Multiple linear regression

Table 4 shows the findings of the multiple linear regression analysis for psychiatric nurses' TI, with WPV, ward atmosphere, and social distance as independent variables. The model was significant [*R*^2^ = 0.193, Δ*R*^2^ = 0.191, *F*_(3, 1333)_ = 106.34, *P* < 0.001]. WPV (β = 0.088, *P* < 0.001), ward atmosphere (β = −0.368, *P* < 0.001), and social distance (β = 0.082, *P* < 0.001) were all significant predictors of turnover intention. Collinearity diagnostics indicated no multicollinearity concerns (all VIF < 2; see Table 2).

**Table 4:** Multiple linear regression (*N* = 1,337).

Variable	β	*t*	*P*	LLCI	ULCI
WPV	0.088	10.37	< 0.001	0.071	0.105
Ward atmosphere	−0.368	−14.23	< 0.001	−0.419	−0.317
Social distance	0.082	8.97	< 0.001	0.064	0.100

WPV, workplace violence.

Dependent variable = turnover intention. *R*^2^ = 0.193, Δ*R*^2^ = 0.191, *F*_(3, 1333)_ = 106.34, *P* < 0.001.

### 3.5 Serial mediating effect

**Figure 3:**
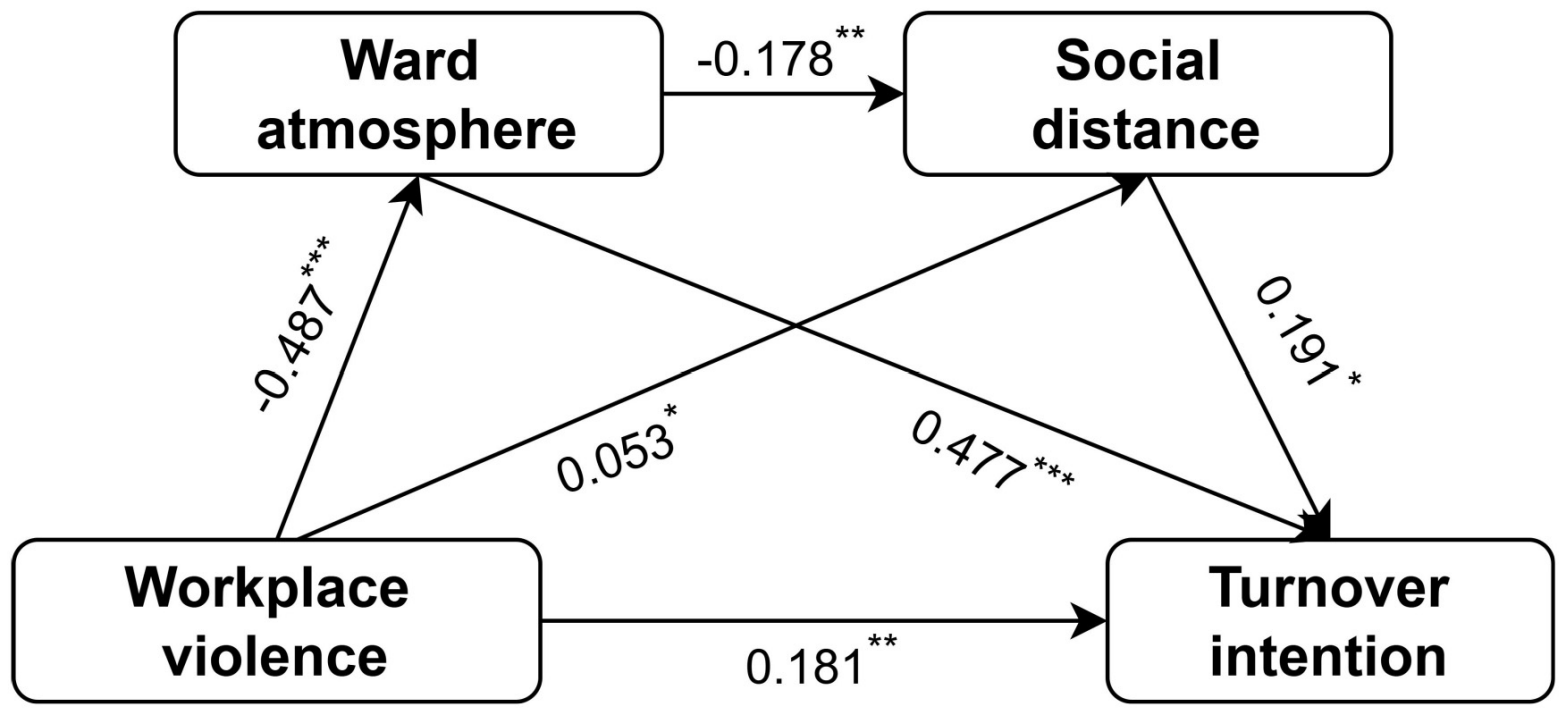
The serial mediating model. *p < 0.05, **p < 0.01, ***p < 0.001.

Table 5 shows an acceptable model fit (*R*^2^ = 0.193, *F* = 106.339, *P* < 0.001). As shown in Figure 3, WPV directly predicted TI among psychiatric nurses (β = 0.181, *P* < 0.001). WPV also indirectly influenced TI through the specific indirect effects of ward atmosphere (β = 0.232, *P* < 0.001) and social distance (β = 0.010, *P* < 0.001). Additionally, the serial mediating effect of ward atmosphere and social distance was significant (β = 0.017, *P* = 0.007). Table 6 indicates that the direct effect accounted for 41.136% of the total effect. Among the indirect effects, the mediating pathway (WPV → ward atmosphere → TI) contributed the highest proportion, reaching 52.727%.

**Table 5:** The serial mediating effects of ward atmosphere and social distance on the relationship between WPV and TI (*N* = 1,337).

Outcome variable	Prediction variable	*R*	*R* ^2^	*F*	β	LLCI	ULCI	*t*
Ward atmosphere	WPV	0.307	0.094	139.026^***^	−0.487^***^	−0.568	−0.406	−11.791
Social distance	WPV	0.345	0.119	90.029^***^	0.053^*^	0.0065	0.990	2.235
	Ward atmosphere				−0.178^**^	−0.208	−0.149	−11.905
TI	WPV	0.439	0.193	106.339^***^	0.181^**^	0.077	0.285	3.406
	Ward atmosphere				−0.477^***^	−0.546	−0.408	−13.549
	Social distance				0.191^*^	0.072	0.311	3.134

LLCI, lower limit of the 95% confidence interval; ULCI, upper limit of the 95% confidence interval; WPV, workplace violence; TI, turnover intention.

^*^P < 0.05, ^**^P < 0.01, ^***^P < 0.001.

**Table 6:** Evaluation of the multiple chain mediating effects of WPV on TI (*N* = 1,337).

Effect	Path	β	SE	LLCI	ULCI	Effect size
Direct effect	WPV → TI	0.181^**^	0.053	0.077	0.285	41.136%
Mediating effect	Total mediating	0.259	0.027	0.207	0.313	58.863%
	WPV → ward atmosphere → TI	0.232^***^	0.025	0.184	0.284	52.727%
	WPV → Social distance → TI	0.010^**^	0.006	0.001	0.024	2.272%
	WPV → ward atmosphere → Social distance → TI	0.017^**^	0.006	0.005	0.029	3.863%
Total effect		0.440^***^	0.055	0.333	0.548	100%

^**^P < 0.01, ^***^P < 0.001.

## 4 Discussion

Grounded in SET, this study examined the mediating mechanisms through which WPV, ward atmosphere, and social distance collectively shape psychiatric nurses' TI. We developed a theoretical framework that elucidates (1) the direct impact of WPV on TI, (2) the buffering role of a positive ward atmosphere in mitigating such effects, and (3) the serial mediation pathway wherein WPV exacerbates social distance, which in turn amplifies TI. By incorporating the Social Exchange Theory (SET)framework, this model significantly enriches the current understanding of the dynamics at play. It provides valuable and practical guidance for organizations seeking to implement targeted interventions that enhance nurse retention within mental health care environments.

### 4.1 The direct effect of WPV on TI

This study confirmed that WPV has a direct positive effect on TI among psychiatric nurses, which is consistent with previous findings (19). The positive correlation between WPV frequency and TI demonstrates a robust, graded association that persists uniformly across nurses, physicians, and therapists (51). WPV has multiple detrimental effects on nursing professionals (52), including poor job satisfaction (53, 75), increased absence at work (54), elevated physiological stress and burnout (55, 56), headache, decreased appetite and insomnia (57). All the detrimental effects can be regarded as the initial resources of negative reciprocity within the social exchange process. When such negative reciprocity reaches a limit, nurses may opt for turnover as a resource preservation strategy to mitigate further self-exhaustion. WPV-exposed nurses report heightened TI and often discourage their offspring from pursuing careers in psychiatry (23). This will further impede the development of the psychiatric nursing workforce in China. Although various interventions to prevent WPV have been implemented (58–60), the incidence rate of WPV remains stubbornly high. Emerging evidence has revealed that different types of WPV exert distinct impacts on psychiatric nurses (19). Future efforts should focus on developing subtype-targeted interventions to enhance the precision and efficacy of prevention strategies.

### 4.2 Ward atmosphere as a mediator

This study confirmed that ward atmosphere mediates the relationship between WPV and TI among psychiatric nurses. Based on the findings, we found that when nurses experience WPV, a positive ward atmosphere mitigates their TI. The experience of WPV not only shapes nurses' perceptions of a negative ward atmosphere (61) but also heightens nurses' awareness of patients (62). When such defensive attitudes become normative, they precipitate a self-reinforcing cycle of interpersonal tension and distrust to create an unfavorable ward atmosphere. A qualitative study suggested that nurses witnessing colleagues experiencing WPV develop anticipatory anxiety about potential WPV, thereby creating a feedback loop that further exacerbates the deterioration of the ward atmosphere (63, 64). Another qualitative study revealed that nurses attributed WPV to patients' pathological conditions, which directed their attention toward therapeutic outcomes rather than preventing WPV (65, 66). This finding suggested a potential positive correlation between WPV and the therapeutic dimension of the ward atmosphere. Future research should explore the differential impacts of WPV across various dimensions of the psychiatric ward atmosphere. However, the increased emphasis on monitoring treatment efficacy may inadvertently escalate nurses' workload, thereby potentially contributing to TI.

If nurses perceive a positive ward atmosphere, they may be more likely to report and actively take measures to reduce the negative impacts caused by WPV (67). This positive perception disrupts the transmission chain of WPV-induced negative reciprocity. Conversely, in negative environments, WPV triggers destructive exchange dynamics between individuals and the environment. Such negative feedback loops propel the ward atmosphere toward further deterioration, ultimately manifesting as elevated TI as nurses seek to prevent resource depletion. The results of this study revealed that the ward atmosphere accounted for the majority of the mediation effect, potentially because atmosphere changes are often contagious within the organization, likely causing a “civility spiral” (68). The emergence of a negative ward atmosphere can trigger detrimental effects among staff members, exacerbating the deterioration of the atmosphere. These findings highlight the urgent need to prioritize psychiatric ward atmosphere management by fostering a positive environment, which reduces staff turnover. A supportive ward atmosphere benefits both nurses and patients, creating a therapeutic setting that sustains ongoing care.

### 4.3 Social distance as a mediator

This study confirmed that social distance mediates the relationship between WPV and TI among psychiatric nurses; when nurses experience WPV, lower social distance toward patients is associated with reduced TI, although the effect was small. Psychiatric nurses' expectations of social distance from patients support the contact hypothesis (69). WPV, as negative contact, intensifies psychiatric nurses' desired social distance from patients, reflecting attitudinal alienation toward psychiatric patients (31). This distance mechanism not only undermines professional identity formation but also contributes to staff turnover (47). These findings extended SET by validating its psychological transaction framework.

### 4.4 Serial mediating effect of ward atmosphere and social distance

These results revealed the serial mediating effect of ward atmosphere and social distance on the relationship between WPV and TI among psychiatric nurses, although the effect size was small. Despite the modest magnitude, this significant serial pathway theoretically supports the “transaction chain” concept of SET (17, 38), demonstrating that negative workplace experiences can sequentially impair environmental perceptions and interpersonal attitudes before influencing turnover intention. The social distance of a certain group of people is determined by the perception of the environment (41). WPV triggers negative feedback between individuals and their environment, including a negative ward atmosphere. This fuels covert transactions that exacerbate social isolation and exclusionary attitudes. As resource loss escalates through this feedback chain, nurses ultimately choose turnover to conserve future resources—further validating SET's “dynamic transaction chain.”

For Hypotheses 3 and 4, the effect sizes were both small. The minimal explanatory power of these pathways reflects the “healthy worker effect” (70). A previous study revealed that medical students may refuse to enter psychiatry departments with greater social distance (71, 72). Our analysis focused exclusively on currently employed psychiatric nurses, excluding individuals who either rejected psychiatry careers or had already quit the profession. The participants included in the study showed relatively small variation in social distance toward psychiatric patients and had a high acceptance rate; most did not intend to resign due to this behavior. This limited variation in key attitudes likely attenuated the observed effect sizes. This underscores the critical necessity of acknowledging, rather than overlooking, the modest effect size. Efforts should intensify social distance education and training while concurrently implementing preventive student-period interventions to mitigate patient alienation and ensure psychiatric workforce stability (73). Meanwhile, based on the multi-foci perspective and psychological exchange of SET, it is essential to consider the role of social distance. This requires us to not only focus on the work environment but also to enhance psychiatric nurses' education regarding their attitudes toward patients and strengthen their professional identity.

### 4.5 Limitations

Several limitations should be acknowledged. First, this study used a cross-sectional design, so causal inferences should be interpreted with caution. Further longitudinal studies are needed to validate these findings. Secondly, turnover intention was assessed using a single item, which may limit the predictive validity of variable relationships and bring potential challenges to multivariate statistical interpretation. Future studies are encouraged to use standardized multi-item scales to improve measurement robustness. Thirdly, the data for this study were derived exclusively from self-report questionnaires. Reliance on self-reported inner feelings introduces potential biases from individual experiences and self-protective response tendencies. Future studies should incorporate diverse data collection methods, including peer nomination and so on. Finally, while ward atmosphere was analyzed as a composite score, the distinct contributions of its three subdimensions were not examined. Future research should examine the unique contributions of each sub-dimension.

## 5 Conclusion

In summary, this study validated the SET framework grounded in emotional theory to explain how WPV directly increased psychiatric nurses' TI, with ward atmosphere and social distance playing independent and sequential mediating roles in this relationship.

Theoretically, this study enriches Social Exchange Theory in psychiatric settings by highlighting the invisible psychological transactions underlying social distance, which serves as a key implicit pathway connecting negative workplace experiences to turnover intention. It also validates the “transaction chain mechanism” and supports the view that organizational environmental exchange precedes individual interpersonal exchange in the workplace. Practically, hospitals should strengthen violence prevention, build a supportive ward atmosphere, reduce nurses' social distance toward patients, and strengthen professional identity to alleviate TI and stabilize the psychiatric nursing workforce. Future longitudinal research is recommended to verify causal relationships.

## Data Availability

The raw data supporting the conclusions of this article will be made available by the authors, without undue reservation.
